# Mothers, Intrinsic Math Motivation, Arithmetic Skills, and Math Anxiety in Elementary School

**DOI:** 10.3389/fpsyg.2017.01939

**Published:** 2017-11-13

**Authors:** Lital Daches Cohen, Orly Rubinsten

**Affiliations:** The Edmond J. Safra Brain Research Center for the Study of Learning Disabilities, Department of Learning Disabilities, University of Haifa, Haifa, Israel

**Keywords:** educational psychology, emotional development, math anxiety, math achievement, intrinsic math motivation

## Abstract

Math anxiety is influenced by environmental, cognitive, and personal factors. Yet, the concurrent relationships between these factors have not been examined. To this end, the current study investigated how the math anxiety of 30 sixth graders is affected by: (a) mother’s math anxiety and maternal behaviors (environmental factors); (b) children’s arithmetic skills (cognitive factors); and (c) intrinsic math motivation (personal factor). A rigorous assessment of children’s math anxiety was made by using both explicit and implicit measures. The results indicated that accessible self-representations of math anxiety, as reflected by the explicit self-report questionnaire, were strongly affected by arithmetic skills. However, unconscious cognitive constructs of math anxiety, as reflected by the numerical dot-probe task, were strongly affected by environmental factors, such as maternal behaviors and mothers’ attitudes toward math. Furthermore, the present study provided preliminary evidence of intergenerational transmission of math anxiety. The conclusions are that in order to better understand the etiology of math anxiety, multiple facets of parenting and children’s skills should be taken into consideration. Implications for researchers, parents, and educators are discussed.

## Introduction

**FIGURE 1:**
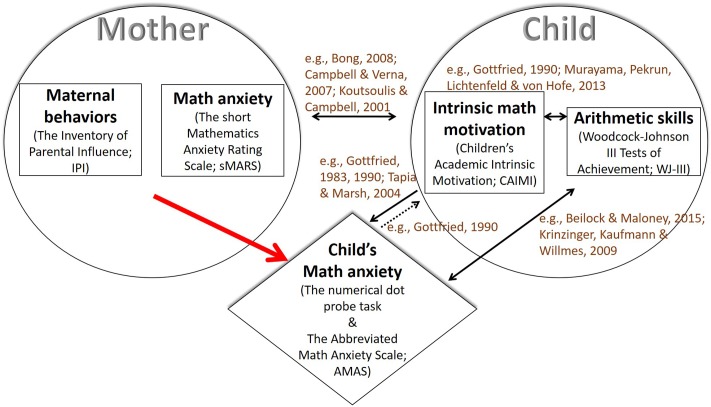
The scientific framework for the present investigation. The rhombus represents the dependent variable in the present study and the red arrow represents the relationship that was the focus of the study. Broken arrows represent weaker relations compared to the bold ones. In brackets is the methodological tool that was used.

In present day society there is a growing reliance on technology and the fields of engineering and mathematics (Ashcraft and Moore, 2009). Yet, many people experience feelings of tension, anxiety, and even fear when engaging in math, a phenomenon known as math anxiety (Beilock and Maloney, 2015). It is important, therefore, to understand math anxiety and to develop ways of reducing its prevalence (Ashcraft and Moore, 2009). One promising line of research is to further explore the role of parents (Dowker et al., 2016), who have been found to have a powerful influence on children’s math achievements and motivation (Koutsoulis and Campbell, 2001). Motivation, in turn, has an important role in mobilizing cognitive resources and mitigating the effects of anxiety on performance (Lyons and Beilock, 2011; Wang et al., 2015). The current study takes up the issue of the role of mothers’ math anxiety, maternal behaviors, children’s motivation, and arithmetic skills in the development of math anxiety (**Figure 1**).

### Math Anxiety: Definition, Prevalence, and Assessment

Math anxiety has been defined by Richardson and Suinn (1972) as a negative emotional reaction to situations involving numbers, calculations, and mathematics that affects the ability to solve problems and manipulate numbers in various academic and daily contexts. Across the Organization for Economic Co-operation and Development (OECD, 2013) countries that participated in the Program for International Student Assessment (PISA), around 30% of 15-year-old students reported feelings of nervousness and helplessness when solving math problems, and 33% acknowledged that they feel tense when faced with math homework. Nonetheless, estimates of the prevalence of math anxiety vary according to the population, the assessment tools, and the classification and assessment criteria (Dowker et al., 2016).

The primary method for evaluating math anxiety is through the assessment of accessible self-representations by using explicit measures, such as the Mathematics Anxiety Rating Scale (MARS; Richardson and Suinn, 1972). Although the reliability of these explicit tools has generally been found to be good (Dowker et al., 2016), identical responses may indicate different levels of subjective anxiety between individuals (Ben-Zeev et al., 2005). For example, men have consistently been found to score lower than women on explicit self-report questionnaires of trait anxiety (e.g., Costa et al., 2001; Egloff and Schmukle, 2004). This could be explained, in part, by the women’s greater inclination to reveal personal attitudes compared to men, rather than by gender differences in anxiety *per se* (Ashcraft, 2002).

On the other hand, implicit tools assess inaccessible cognitive structures. Since the use of these measures is less common than explicit tools, it is necessary to introduce the implicit assessment of math anxiety. It has been shown that affective traits can be activated automatically after seeing a salient affective stimulus (for review see Rubinsten, 2015) and that they influence cognitive, behavioral, and emotional processes (e.g., Giner-Sorolla et al., 1999). For example, individuals with different anxiety problems tend to display a differential attentional allocation toward threat-related information relative to neutral stimuli, a phenomenon known as attentional bias (MacLeod et al., 1986; Bar-Haim et al., 2007). Attentional bias toward threat-related information has been found to play a key role in the development and persistence of anxiety disorders (Mathews and MacLeod, 2002).

**FIGURE 2:**
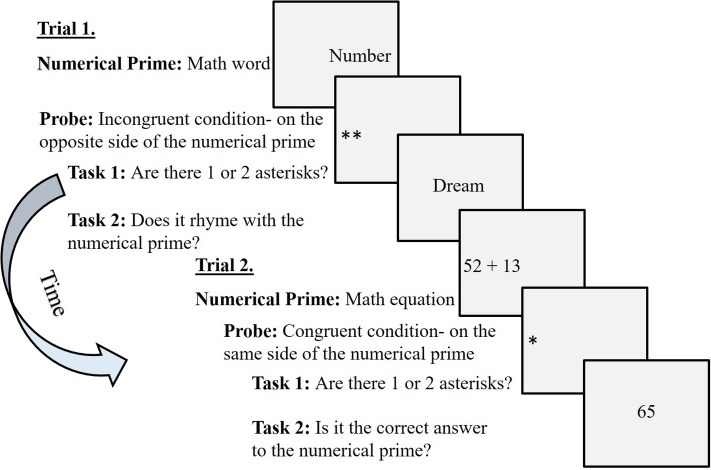
Examples of trials in the numerical dot-probe task (Rubinsten et al., 2015).

Therefore, measures of attentional bias can be used as a cognitive tool for implicit assessment. One experimental paradigm for studying attentional bias toward threat-related information is the dot-probe task (MacLeod et al., 1986), which has been found a reliable tool for the diagnosis and treatment of general anxiety (e.g., Baert et al., 2010). In the numerical version of the canonical dot-probe task (Rubinsten et al., 2015), a probe (either one or two asterisks) appears on one side of the computer screen that is *congruent* or *incongruent* with the location of math-related (a math word, such as “number,” or a math equation, such as 52 + 13) or neutral prime stimuli (a word with neutral emotional tone, such as “picture”). First, participants must discriminate the probe’s identity (one or two asterisks) as fast as possible. Then, a word (after a math or neutral word prime) or a number (after a math equation prime) appears in the center of the computer screen. Participants are requested to decide whether the word rhymes with the previously presented math or neutral word or whether the number is the correct solution to the previously presented math equation (for illustration of the trials see **Figure 2**).

Consistent with previous findings that indicated attentional bias toward threat-related stimuli among high-anxious individuals in the dot-probe task (Koster et al., 2005, 2006), Rubinsten et al. (2015) found attentional bias toward math-related information among high math anxious university students in the numerical dot-probe task. This latter attentional bias was reflected in the *congruency effect*, which was manifested as faster responses to a probe in the congruent trials as compared to the incongruent trials. These findings suggest that the cognitive system of math-anxious individuals electively favors to process math-related stimuli, which are cognitively interpreted as threatening and with a negative valence (Rubinsten and Tannock, 2010; Rubinsten et al., 2012).

Hence, as an implicit measure of children’s math anxiety in the current study, the numerical dot-probe task was administered. Specifically, the tendency of math-anxious individuals to display attentional bias toward math-related stimuli was examined. The study of attentional bias is particularly relevant for developmental research and pedagogical practices because it impairs attentional control and enhances susceptibility to distractions (Suárez-Pellicioni et al., 2014) or raises the inhibition of anxiety-related responses (Pletzer et al., 2015). Thus, attentional bias toward math-related stimuli could reduce efficiency in math problem solving (Rubinsten et al., 2015).

### Math Anxiety and Achievements

Math anxiety has consistently been shown to be negatively related to math achievements (Hembree, 1990; Ma, 1999). When faced with a math task, math-anxious individuals tend to have worries and intrusive thoughts about the situation and its consequences that may distract their attention and disrupt their thinking processes (Chang and Beilock, 2016). These distractions consume valuable attentional resources of the working memory (Ashcraft and Kirk, 2001; Maloney and Beilock, 2012), which is a limited short-term memory system that enables inhibition of irrelevant information and integrates, stores, and manipulates the information relevant to the task at hand (Baddeley, 2000).

However, poor math performance may be the cause of math anxiety (Ma and Xu, 2004). Recently, math anxiety has been found to be associated with basic numerical skills, such as simple counting (Maloney et al., 2010; Rubinsten and Tannock, 2010; Rubinsten et al., 2012). Furthermore, children with diagnosed mathematical disabilities have been found to have more math anxiety compared to a control group (Rubinsten and Tannock, 2010; Passolunghi, 2011). Actually, math anxiety may be both the cause and the result of low achievements (e.g., Ashcraft and Moore, 2009). Beyond the direct negative impact of math anxiety on performance, avoidance behavior caused by math anxiety (Hembree, 1990; Ashcraft, 2002; Ashcraft and Ridley, 2005) will most probably lead to a vicious cycle in which avoidance of math will create gaps in learning which in turn exacerbate emotional problems (Krinzinger et al., 2009). Although the directionality of the relationship between math anxiety and achievements is open to debate, the negative correlation between them has been found as early as first and second grade (Ramirez et al., 2013).

### The Development of Math Anxiety

Recent findings have shown that children as young as first-grade self-report varying levels of math anxiety (Young et al., 2012; Ramirez et al., 2013; Maloney et al., 2015). However, in the fourth and fifth grade, the emergence of math anxiety is clearly recognizable (Ashcraft and Moore, 2009). In his meta-analysis, Hembree (1990) indicated that levels of math anxiety rise between elementary and high school, with a significant increase between ninth and tenth grades and a plateau thereafter. Little is known about the causes underlying this development, which may be environmental, such as negative experiences (Harper and Daane, 1998), cognitive, such as poor math abilities (Newstead, 1998), or personal, such as low motivation (Tapia and Marsh, 2004).

### Intrinsic Math Motivation

Motivation is part of the individual’s goals and beliefs and it determines the degree of involvement in specific situations (Ames, 1992). In line with the Self-Determination Theory (Deci and Ryan, 1985), it is conventional to distinguish between intrinsic academic motivation that drives the individual to learn for his or her own sake, extrinsic academic motivation, in which external feedback motivates learning, and amotivation, which refers to a lack of intention to act (Gottfried, 1985, 1990). Intrinsic academic motivation has been found to have good reliability, validity, and significance for learning (Gottfried, 1983, 1985). For instance, in a longitudinal study from ages 7–9 years, Gottfried (1990) found that higher levels of intrinsic math motivation are associated with poorer performance on standardized tests. Moreover, intrinsic math motivation predicted achievements and explained growth in achievements from the fifth to tenth grade (Murayama et al., 2013). This relationship appears to be reciprocal because achievements have been found to influence intrinsic math motivation from elementary to high school (Garon-Carrier et al., 2016).

### Intrinsic Math Motivation and Math Anxiety

Individuals with high math anxiety often express negative attitudes toward math and tend to have low math motivation (Hembree, 1990). Indeed, a significant negative correlation has been found between intrinsic math motivation and math anxiety (Gottfried, 1990). These attitudes and beliefs possibly cause learners to invest less effort and time in math learning (Maloney et al., 2015). Therefore, intrinsic math motivation and math anxiety together improve the prediction of math performance compared with either one alone (Lyons and Beilock, 2011). Specifically, math motivation can help diminish avoidance behavior and overcome anxiety-related responses (Chang and Beilock, 2016). Recently, a negative linear relationship was found between math anxiety and achievements in adolescents and adults with low intrinsic math motivation, whereas an inverted-U curvilinear relationship was observed in more motivated students (Wang et al., 2015).

Unfortunately, several studies have shown a decline in intrinsic math motivation from elementary to high school (Corpus et al., 2009; Garon-Carrier et al., 2016). In fact, intrinsic math motivation at all ages from age 9 years predicted the levels of intrinsic math motivation in subsequent ages until age 16 years (Gottfried et al., 2001). The factors underlying this development are not clear (Spinath and Spinath, 2005). According to the Self-Determination Theory (Deci and Ryan, 1980, 1985), parents can contribute to the development of intrinsic academic motivation by positive feedback and encouragement of autonomous behaviors. On the other hand, parenting with more controlling aspects, such as the use of clear rules and surveillance of homework, will undermine intrinsic motivation.

### Parental Behaviors and Attitudes toward Math

Perceived expectations, pressure, and support from parents may cause children to feel confident or helpless and shape their interests (Bong, 2008) and attitudes toward school (Koutsoulis and Campbell, 2001). Indeed, research has established a positive contribution of parental support (Koutsoulis and Campbell, 2001; Campbell and Verna, 2007; Bong, 2008; Fan and Williams, 2010) as well as a negative contribution of parental pressure (Ginsburg and Bronstein, 1993; Bong, 2008) to intrinsic math motivation. While a psychologically supportive atmosphere at home represents parents who help their children develop better attitudes and beliefs toward their academic abilities, a pressure environment suggests a demanding parent who applies pressure to maintain high achievements (Campbell, 1994; Campbell and Verna, 2007). Help with schoolwork and monitoring by parents have also been found to reduce children’s intrinsic math motivation (Campbell and Verna, 2007). These findings support the Self-Determination Theory (Deci and Ryan, 1980, 1985) mentioned above.

Parents also have a prominent role in the development of math anxiety by being children’s primary socializers and role models (Maloney et al., 2015; Chang and Beilock, 2016). Thus, parental attitudes toward mathematics and parents’ educational level are associated with levels of math anxiety among children (Turner et al., 2002; Scarpello, 2007). For instance, when math-anxious parents report frequently helping with students’ math homework, their children’s math-learning processes during the school year are reduced and their levels of math anxiety at the end of the year are higher (Maloney et al., 2015). Besides “transferring” their negative conception of math to their children, parents can also elevate their children’s math anxiety through the use of certain parental behaviors.

The relationship between parental behaviors and children’s anxiety-related behaviors is also well-documented (e.g., Wood et al., 2003; McLeod et al., 2007). For example, Quach et al. (2015) indicated a significant positive correlation between parental pressure and symptoms of anxiety, while parental support had a negative correlation with these symptoms. However, this latter correlation disappeared or became positive when parental pressure was taken into account. In the case of math anxiety, Roberts and Vukovic (2011) found a negative correlation between parental involvement and math anxiety, but only five items were used to assess parental involvement in children’s math learning. By reducing the level of math anxiety, parental involvement raised second graders’ performance on higher levels of math, such as problem-solving (Vukovic et al., 2013).

### The Current Study

Although the construct of math anxiety has received increasing attention in recent years (Dowker et al., 2016), the simultaneous relationship between mothers’ math anxiety, maternal behaviors, children’s intrinsic math motivation, arithmetic skills, and math anxiety has not been examined. To address this existing knowledge gap, mothers’ perceptions of five parental practices (pressure, psychological support, help, press for intellectual development, and monitoring/time management) as well as mothers’ self-reports of their math anxiety and math skills in high school and at present were assessed. Only mothers participated in the study for reducing data variability and because they are typically serve as children’s primary caregivers (Sayer et al., 2004) and tend to be more involved than fathers in children’s schooling (Grolnick and Slowiaczek, 1994). In addition, their sixth-grade children’s intrinsic motivation toward school learning in general and math in particular, arithmetic skills, and math anxiety were evaluated. There were two reasons for recruiting this age group. First, math anxiety can be clearly identified at this age (Ashcraft and Moore, 2009). Second, by this age, a substantial degree of intrinsic math motivation has developed and predicts levels of intrinsic math motivation at subsequent ages (Gottfried et al., 2001).

The reviewed literature (e.g., Gottfried, 1990; Murayama et al., 2013; Garon-Carrier et al., 2016) led us to hypothesize, first, that intrinsic math motivation would have a positive correlation with arithmetic skills and that these two constructs will have negative correlations with math anxiety. Secondly, consistent with the Self-Determination Theory (Deci and Ryan, 1980, 1985) and previous findings (e.g., Campbell and Verna, 2007; Bong, 2008; Chang and Beilock, 2016), maternal practices that include the more rigid aspects (i.e., pressure, help, press for intellectual development, and monitoring) were assumed to be associated with less intrinsic math motivation, poorer arithmetic skills, and higher levels of math anxiety. Thirdly, it was hypothesized that mothers who were more math-anxious would have children who show less intrinsic math motivation, poorer arithmetic skills, and higher levels of math anxiety. Finally, considering the distinct aspects of math anxiety that will be assessed by explicit (i.e., the AMAS self-report questionnaire) and implicit measures (i.e., the numerical dot-probe task), it would be reasonable to assume that different factors will predict inaccessible cognitive structures as compared to accessible self-representations of children’s math anxiety. The uniqueness of the current study lies in the use of explicit and implicit measures of children’s math anxiety. The use of implicit assessment through the numerical dot-probe task enabled us to manipulate difficulty levels by presenting various math-related stimuli. In this way, the influence of different environmental, cognitive, and personal factors on children’s math anxiety was comprehensively and rigorously examined.

## Materials and Methods

### Participants

Participants included 30 sixth graders (*M* = 11.4-years-old, *SD* = 0.46 years), 13 girls and 17 boys, and their mothers (*M* = 42.7-years-old, *SD* = 4.10 years). There were no significant differences between genders in the variables examined.

### Measures

#### Maternal Behaviors

The 52-item well-established Inventory of Parental Influence (IPI; Campbell, 1994; Campbell and Verna, 2007) was used to identify mothers’ perceptions of five family processes: pressure (i.e., a demanding parent who applies pressure to maintain high achievements; 13 items); psychological support (i.e., a psychologically supportive atmosphere at home; 12 items); help (i.e., a parent who devotes the time required to help his or her child with schoolwork; 10 items); press for intellectual development (i.e., a parent who emphasizes the importance of intellectual resources; 9 items); and monitoring (i.e., a parent who sets rules for time management; 8 items). In the first two family process scales, responders express their degree of agreement with each statement on a five-point Likert scale (1 = *strongly disagree*, 2 = *disagree*, 3 = *uncertain*, 4 = *agree*, 5 = *strongly agree*). In the next three scales, respondents are asked to specify the frequency of various family practices on a five-point Likert scale (1 = *never*, 2 = *rarely*, 3 = *sometimes*, 4 = *usually*, 5 = *always*). The instrument was translated by the authors into Hebrew (forward translation) and then from Hebrew back into English (back translation) to ensure the validity of the translation. Using Cronbach’s alpha, reliability for the IPI was found to be 0.83. All analyses were conducted on the sum of the items for each component. Higher scores indicated greater maternal influence.

#### Mothers’ Math Anxiety

A Hebrew translated and modified version of the short Mathematics Anxiety Rating Scale (sMARS; Alexander and Martray, 1989) was used to evaluate mothers’ math anxiety. The instrument was translated by the authors into Hebrew (forward translation) and then from Hebrew back into English (back translation) to ensure the validity of the translation. The sMARS is a 25-item version of the widely used 98-item MARS (Richardson and Suinn, 1972). It focuses on the three factors that were most salient when a factor analysis was conducted on the MARS: math test anxiety (15 items), numerical task anxiety (5 items), and math course anxiety (5 items). The coefficient alpha for the sMARS was found to be 0.96. Mothers rated the degree of anxiety they feel during different everyday and formal situations on a five-point Likert scale (1 = *not at all*, 2 = *a little*, 3 = *a fair amount*, 4 = *much*, 5 = *very much*). The modification of the instrument involved changing items to the past tense and instructing respondents to refer to previous math experiences in academic settings when answering the questionnaire. All analyses were performed on the sum of all the items and the items of each factor separately, with higher scores reflecting an increased level of anxiety.

#### Mothers’ Math Skills

An estimation of mothers’ math skills was obtained by asking them to rate their skills in math in high school and at present on a seven-point Likert scale (1 = *very low*, 2 = *low*, 3 = *low to moderate*, 4 = *moderate*, 5 = *moderate to high*, 6 = *high*, 7 = *very high*) in order to obtain as accurate an estimation as possible.

#### Socioeconomic Status

Mothers’ occupation was scaled by the International Socioeconomic Index (ISEI; Ganzeboom and Treiman, 1996), which ranges from 16 to 90 based on the International Standard Classification of Occupation 1988 (ISCO-88). For each occupation, ISEI scores reflected the weighted averages of standardized measures of income and education such that higher scores indicated a higher socioeconomic status (Ganzeboom and Treiman, 1996).

#### Children’s Intrinsic Math Motivation

A Hebrew translated version of the Children’s Academic Intrinsic Motivation Inventory (CAIMI; Gottfried, 1986) was used to assess children’s intrinsic academic motivation. In order to ensure the validity of the translation, the questionnaire was translated into Hebrew (forward translation) and then from Hebrew back into English (back translation). The CAIMI is a psychometrically well-validated 122-item self-report inventory that contains five subscales, four of which measure intrinsic motivation in the subject areas of reading, math, social studies, and science, with the fifth measuring intrinsic motivation as a general orientation toward school learning. Participants often do not know the meaning of the term social studies. Therefore, this subject area was omitted. Each of the subject area subscales contain 26 items with the same stem and a separate response for each subject. For 24 items, the respondents express their degree of agreement with each statement on a five-point Likert scale (1 = *strongly disagree*, 2 = *disagree*, 3 = *uncertain*, 4 = *agree*, 5 = *strongly agree*). The remaining two items require a forced choice between an intrinsic and non-intrinsic alternative. The general subscale contains 18 items which are similar in content to those in the subject area subscales. In order to balance the items, high intrinsic motivation is indicated by agreement in approximately half of them, and by disagreement in the other half. The total score of each subscale was obtained by summing the items of each factor separately. Raw scores were converted to normalized *T*-scores. Internal consistency reliability of the CAIMI was 0.97.

#### Children’s Math Anxiety

Children’s math anxiety was evaluated through both explicit and implicit measures. As *an explicit tool*, a Hebrew translated and adjusted form of the well-known Abbreviated Math Anxiety Scale (AMAS; Hopko et al., 2003) was used. The instrument was translated by the authors into Hebrew (forward translation) and then from Hebrew back into English (back translation) to ensure the validity of the translation. The AMAS is a nine-item self-report questionnaire found to be as effective as the longer Math Anxiety Rating Scale (Hopko, 2003). Each item consists of a statement describing an event and participants indicate how anxious it would make them on a five-point Likert scale (1 = *never*, 2 = *rarely*, 3 = *sometimes*, 4 = *usually*, 5 = *always*). The modification of the AMAS for primary-school students involved changing certain wordings, such as “*fear*” instead of “*anxious*.” Scores on the AMAS range from 9 to 45, with a higher score indicating a higher level of math anxiety. Cronbach’s alpha for the AMAS in the current sample was 0.77.

As *an implicit tool*, the numerical dot-probe task (Rubinsten et al., 2015) was used. This is a numerical version of the canonical dot-probe task (MacLeod et al., 1986) that contains a total of 36 trials (for illustration of the trials see **Figure 2**). At the beginning of each trial, a white colored square shaped fixation point was presented for 750-ms. Then, a blank screen was presented for 100-ms, followed by a prime that appeared on one side of the screen for 1000-ms. The prime stimuli could be either math-related (a math equation, such as 52 + 13, or a math word, such as “number”) or neutral (a word with neutral valence, such as “picture”). There were four different math equation levels: (a) a single digit (e.g., 6 ÷ 2); (b) double digit (e.g., 46 + 23); (c) triple digit (e.g., 536 - 268); and (d) a power equation (e.g., 4^3^ × 8^2^). All of the math equations included simple addition, subtraction, multiplication, or division operations.

After the appearance of the prime, there was an inter stimulus interval (ISI) of 100–150-ms. Next, a probe (either one or two asterisks) appeared on the left or right side of the computer screen, *congruent* or *incongruent* with the location of the previously presented prime. To avoid visual bias, the exact location of the probe was chosen randomly. Participants were instructed to discriminate the probe’s identity as fast as possible by pressing the matching key on the keyboard (“1” for one asterisk or “2” for two asterisks). The probe was displayed until the participant responded or for 3000-ms. After responding, in cases of math equation prime trials, a number appeared in the center of the screen, and participants were asked to decide whether it is the correct answer to the previously presented equation by pressing a matching key on the keyboard (“1” for correct answer and “2” for wrong answer). In cases of math or neutral word prime trials, another word appeared in the center of the screen, and participants had to decide whether it rhymes with the previously presented word by pressing a matching keyboard key (“1” for correct answer and “2” for wrong answer). In half the trials, the answer was correct. This task was designed to ensure that participants processed the prime meaningfully. Following the participant’s decision or after 4000-ms, a black screen was displayed for 1500-ms, and then the next trial began.

The task consisted of six blocks, each containing one sample of each stimuli type (four equation levels, math related and neutral words) and followed by a 1-min break. During the break, an aquarium film appeared on the computer screen in order to avoid ongoing stress levels. Overall, the task lasted about 45-min. As mentioned earlier, the *congruency effect*, which is the difference between reaction time in the incongruent and congruent conditions, was calculated in each of the six prime types. A higher congruency effect indicated the tendency of math-anxious individuals to display attentional bias toward math-related stimuli (Rubinsten et al., 2015). The trials analyzed included only those in which the probe was correctly identified.

#### Children’s Arithmetic Skills

Children’s arithmetic skills were measured using two subtests from the Woodcock–Johnson III (WJ-III) Tests of Achievement: Calculation and Math Fluency (Woodcock et al., 2001). The calculation subtest measured the ability to perform mathematical computations in traditional written format. In this subtest, participants solve increasingly difficult calculation problems without a time limit. The math fluency subtest assessed automaticity with basic arithmetic facts and required children to quickly and accurately complete simple arithmetic problems within a 3-min time limit. These arithmetic computations include simple addition, subtraction, and multiplication operations presented in traditional written format. For each subtest, the number of correctly completed computations was totaled and converted to a standard score with equal intervals.

### Procedure

Prior to data collection, permission was obtained from parents and students. Parents were informed that participation in the study involved filling out questionnaires in groups and completing a computerized task individually. In addition, parents were informed that identifying data would be used for follow-up purposes and would be accessible only to the researchers until the conclusion of the study. Mothers received the questionnaires at a parent–teacher conference; they completed the questionnaires and returned them to the authors via their children. The questionnaires were organized in the following order: IPI, sMARS, and self-ratings of their math skills in high school and at present. Participating children were invited to a single session in a quiet room at school to complete the self-rating questionnaires, which were administered by the researchers in groups in the following fixed order: CAIMI, AMAS, and the math fluency and calculation subtests. Children were instructed on how to fill out the questionnaires and encouraged to request assistance if items are hard to understand. In the second meeting, held at the most a week later in the same room, the computerized numerical dot-probe task was administered individually. This task was programmed in E-Prime and the participants sat approximately 60 cm from the screen. To note, the experiment and overall procedure were approved by the University of Haifa ethics committee.

### Statistical Analyses

To assess the hypotheses, intercorrelations were calculated between mothers’ and children’s variables. The utility of different variables to predict children’s math anxiety was further examined, as measured explicitly (i.e., by the AMAS self-report questionnaire) and implicitly (i.e., by the numerical dot-probe task), using hierarchical linear regressions. These analyses included variables that were significantly correlated with the explicit and implicit measures of children’s math anxiety. All regressions had the same structure; personal factors were entered in the first steps and maternal factors in the following steps. This structure is based on previous evidence indicating the primary influence of parental behaviors on children’s attributes and behaviors (Gonzalez and Wolters, 2006; Fan and Williams, 2010) which, in turn, affect emotional reaction to situations involving math (Lyons and Beilock, 2011; Chang and Beilock, 2016).

## Results

The final sample consisted of 27 mothers (*M* = 42.7-years-old, *SD* = 4.16 years) and their sixth-grade children (*M* = 11.42-years-old, *SD* = 0.47 years), 12 girls and 15 boys. Three additional participants were removed from analyses because they had less than 90% accuracy rates in the probe identification task (i.e., task 1 in the numerical dot-probe task; see **Figure 2**). Likewise, the power equation trials were not analyzed because mean accuracy rates in the second task in these trials (i.e., for deciding whether the number presented is the correct solution of these equations; see **Figure 2**) were significantly lower than 50% [*t*(26) = -2.75, *p* < 0.025]. It may have been too difficult or complicated for them to solve mentally. Descriptive statistics of the research variables are presented in **Table 1**.

**Table 1:** Descriptive statistics of the research variables.

	*M*	*SD*
**Children’s variables**		
(1) Math fluency	101.22	13.13
(2) Calculation	108.07	9.01
(3) AMAS	22.77	7.59
(4) Intrinsic reading motivation	-1.06	1.30
(5) Intrinsic math motivation	-0.41	1.21
(6) Intrinsic science motivation	-0.36	1.35
(7) Intrinsic general motivation	-0.76	1.15
(8) Congruency effect – single digit	-47.02	396.02
(9) Congruency effect – double digit	-131.42	364.77
(10) Congruency effect - triple digit	-74.52	338.80
(11) Congruency effect - math word	-27.07	154.49
(12) Congruency effect - neutral word	-5.64	182.27
**Mothers’ variables**		
(13) Years of schooling	15.07	2.26
(14) ISEI	59.40	17.69
(15) Math skills in high school	5.26	1.45
(16) Math skills at present	4.78	1.36
(17) Pressure	42.03	5.01
(18) Psychological support	37.03	4.35
(19) Help	34.92	5.13
(20) Press for intellectual development	28.40	7.74
(21) Monitoring	25.00	5.72
(22) Math test anxiety	45.25	13.95
(23) Numerical task anxiety	13.07	5.99
(24) Math course anxiety	12.33	4.72
(25) Math anxiety	70.66	22.21


*AMAS, the Abbreviated Math Anxiety Scale (Hopko et al., 2003); congruency effect, the difference between reaction time in the incongruent and congruent conditions in the numerical dot-probe task (Rubinsten et al., 2015); ISEI, the International Socioeconomic Index (Ganzeboom and Treiman, 1996).*

### The Role of Intrinsic Math Motivation and Arithmetic Skills

**FIGURE 3:**
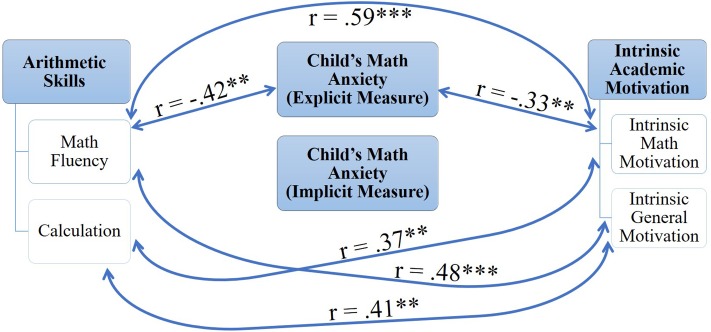
A schematic summary of the correlations between children’s variables. ^∗∗^*p* < 0.05, ^∗∗∗^*p* < 0.01 (one-tailed).

In order to examine whether lower levels of math anxiety are associated with greater ability and motivation, correlations between the children’s measures were calculated (**Table 2**). **Figure 3** displays a schematic summary of these correlations. Children’s math anxiety was assessed through the AMAS questionnaire and the congruency effect (i.e., the difference between reaction time in the incongruent and congruent conditions) in the five prime types on the numerical dot-probe task.

**Table 2:** Pearson correlation matrix of children’s variables.

	1	2	3	4	5	6	7	8	9	10	11	12
(1) Math fluency	-											
(2) Calculation	0.76**	-										
(3) AMAS	-0.42*	-0.28	-									
**Intrinsic motivation**												
(4) Reading	0.28	0.24	-0.27	-								
(5) Math	0.59**	0.37*	-0.33*	0.58**	-							
(6) Science	0.38*	0.31	-0.21	0.48**	0.63**	-						
(7) General	0.48**	0.41*	-0.25	0.48**	0.86**	0.61**	-					
**Congruency effect**												
(8) Single digit	0.17	0.02	-0.19	0.31	0.15	0.03	0.14	-				
(9) Double digit	-0.09	0.05	0.14	-0.17	0.01	0.04	0.16	-0.31	-			
(10) Triple digit	-0.03	0.13	-0.22	-0.29	-0.09	-0.10	0.03	0.13	0.00	-		
(11) Math word	0.18	-0.13	-0.21	0.23	0.04	0.01	-0.21	-0.05	-0.28	-0.29	-	
(12) Neutral word	-0.28	-0.01	-0.04	0.34*	-0.08	0.24	0.06	0.12	0.20	0.07	-0.26	-


*AMAS, the Abbreviated Math Anxiety Scale (Hopko et al., 2003); congruency effect, the difference between reaction time in the incongruent and congruent conditions in the numerical dot-probe task (Rubinsten et al., 2015). ^∗^*p* < 0.05, ^∗∗^*p* < 0.01 (one-tailed).*

As predicted in the first hypothesis, significant positive correlations were found between intrinsic math motivation and measures of arithmetic skills (for math fluency *r* = 0.59, *p* < 0.01; for calculation *r* = 0.37, *p* < 0.05). Moreover, intrinsic math motivation (*r* = -0.33, *p* < 0.05) and math fluency (*r* = -0.42. *p* < 0.025) were significantly negatively correlated with explicitly measured math anxiety (i.e., by the AMAS self-report questionnaire). The congruency effect in the math-related trials, on the other hand, which constitute an implicit measure of math anxiety, was not related to children’s intrinsic math motivation (*p*s between 0.21 and 0.47) and arithmetic skills (*p*s between 0.17 and 0.45).

The results also indicate the salience of math in the construct of intrinsic motivation toward school learning. Intrinsic motivation as a general orientation toward school learning had a higher correlation with intrinsic math motivation (*r* = 0.86, *p* < 0.001) than with intrinsic motivation in the subject areas of reading (*r* = 0.48, *p* < 0.01) and science (*r* = 0.61, *p* < 0.001). Similarly, intrinsic motivation toward school learning was significantly positively correlated with arithmetic skills, as assessed by the math fluency (*r* = 0.48, *p* < 0.01) and calculation (*r* = 0.41, *p* < 0.025) subtests.

Critically, correlations between explicit and implicit measures of math anxiety were found to be non-significant (*p*s between 0.13 and 0.23). Here, it should be noted that Rubinsten et al. (2015) found a significant positive correlation (*r* = 0.40, *p* < 0.05) between the congruency effect in math-related trials and the Hebrew translated computerized version of the MARS-R (Plake and Parker, 1982), which is a shortened version of the MARS questionnaire (Richardson and Suinn, 1972). Possibly, different explicit tools appraise distinct accessible self-representations of math anxiety. Another explanation may be the relatively small sample in the current study.

### The Role of Parents

**FIGURE 4:**
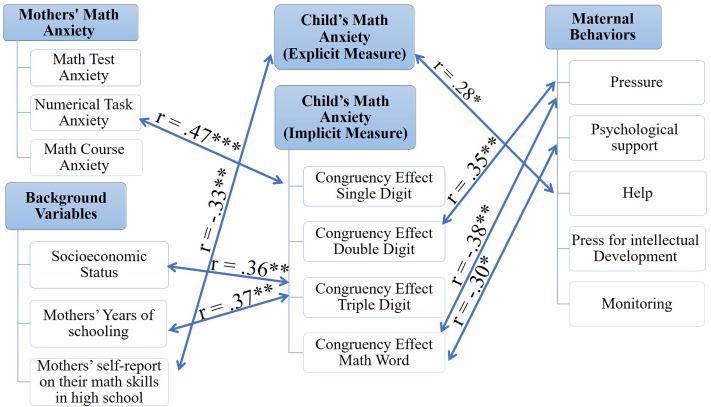
A schematic summary of the correlations between mothers’ variables and children’s math anxiety. ^∗^*p* ≤ 0.07, ^∗∗^*p* < 0.05, ^∗∗∗^*p* < 0.01 (one-tailed).

**FIGURE 5:**
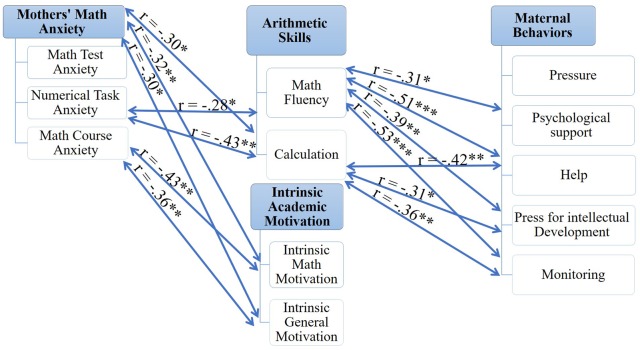
A schematic summary of the correlations between mothers’ variables and children’s arithmetic skills and intrinsic academic motivation. ^∗^*p* ≤ 0.07, ^∗∗^*p* < 0.05, ^∗∗∗^*p* < 0.01 (one-tailed).

Another aim of the current investigation was to examine whether maternal behaviors and mothers’ math anxiety are related to children’s intrinsic math motivation, arithmetic skills, and math anxiety. To this end, a Pearson correlation analysis was used (**Table 3**). A schematic summary of these correlations is presented in **Figures 4** and **5**.

**Table 3:** Pearson correlation matrix between mothers’ and children’s variables.

	YOS	ISEI	MS-HS	MS-T	Pre	Sup	Help	PressID	Mon	MTA	NTA	MCA	MA
Math fluency	0.06	-0.07	0.02	-0.05	-0.19	-0.31	-0.51**	-0.39*	-0.53**	-0.12	-0.28	-0.29	-0.22
Calculation	0.14	0.12	0.18	-0.00	-0.09	-0.22	-0.42*	-0.31	-0.36*	-0.23	-0.43*	-0.17	-0.30
AMAS	-0.09	-0.19	-0.33*	-0.10	0.00	0.21	0.28	0.19	0.23	0.13	0.16	0.19	0.17
**Intrinsic motivation**			
Reading	-0.17	-0.14	0.10	0.17	0.01	-0.17	0.04	0.26	0.09	0.16	0.09	0.04	0.13
Math	-0.02	-0.02	0.19	0.13	-0.00	-0.18	-0.01	0.21	-0.01	-0.25	-0.29	-0.43*	-0.32*
Science	0.28	-0.01	0.17	0.14	-0.08	-0.29	0.18	0.16	0.07	0.00	-0.08	-0.07	-0.03
General	-0.11	0.07	0.24	0.11	0.02	-0.05	0.12	0.23	-0.01	-0.27	-0.22	-0.36*	-0.30
**Congruency effect**			
Single digit	-0.12	-0.11	0.05	0.02	0.15	0.12	-0.01	-0.01	-0.06	0.10	0.47**	-0.02	0.18
Double digit	0.02	0.20	0.26	-0.05	0.35*	-0.02	-0.03	-0.13	-0.19	-0.14	0.04	0.18	-0.04
Triple digit	0.37*	0.36*	0.07	-0.26	-0.11	0.06	0.19	-0.06	0.06	-0.21	-0.18	-0.09	-0.20
Math word	0.03	-0.19	-0.13	-0.02	-0.38*	-0.30	-0.22	-0.10	-0.08	0.11	0.03	-0.05	0.07
Neutral word	0.06	-0.01	0.17	0.20	0.15	-0.02	0.44*	0.41*	0.31	0.08	0.22	0.24	0.16


*YOS, mothers’ years of schooling; ISEI, the International Socioeconomic Index (Ganzeboom and Treiman, 1996); MS-HS, mothers’ self-reports of their math skills in high school; MS-T, mothers’ self-reports of their math skills at present; Pre, maternal pressure; Sup, maternal psychological support; PressID, maternal press for intellectual development; Mon, maternal monitoring; MTA, mothers’ math test anxiety; NTA, mothers’ numerical task anxiety; MCA, mothers’ math course anxiety; MA, mothers’ math anxiety; AMAS, the Abbreviated Math Anxiety Scale (Hopko et al., 2003); Congruency Effect, the difference between reaction time in the incongruent and congruent conditions in the numerical dot-probe task (Rubinsten et al., 2015). ^∗^*p* < 0.05, ^∗∗^*p* < 0.01 (one-tailed).*

First, parenting practices which may be associated with lower intrinsic math motivation and achievements and higher levels of math anxiety were studied. Although correlations between maternal behaviors and intrinsic math motivation were found to be non-significant (*p*s between 0.13 and 0.48), higher levels of maternal behaviors were correlated with poorer arithmetic skills. Lower scores in the math fluency subtest were associated with higher levels of maternal help (*r* = -0.51, *p* < 0.01), press for intellectual development (*r* = -0.39, *p* < 0.025), and monitoring (*r* = -0.53, *p* < 0.01). Math fluency was also marginally significantly negatively related to psychological support (*r* = -0.31, *p* = 0.05). Likewise, the calculation subtest’s scores were negatively correlated with maternal help (*r* = -0.42, *p* < 0.025) and monitoring (*r* = -0.36, *p* < 0.05) and marginally significantly negatively related to press for intellectual development (*r* = -0.31, *p* = 0.05).

Furthermore, parental practices were significantly correlated with children’s implicitly measured math anxiety. Higher levels of maternal pressure were associated with a larger congruency effect in the double-digit trials (*r* = 0.35, *p* < 0.05) and with a smaller effect in the math word trials (*r* = -0.38, *p* < 0.025). A larger congruency effect represented an attentional bias toward math-related stimuli, as manifested in faster responses to a probe presented in the same location (congruent trials) of a numerical prime as compared to the response time when the probe and numerical prime appeared in different locations (incongruent trials). Maternal psychological support was also negatively correlated with the congruency effect in the math word trials, but only marginally (*r* = -0.30, *p* = 0.06). Interestingly, socioeconomic status (*r* = 0.36, *p* < 0.05) and mothers’ years of schooling (*r* = 0.37, *p* < 0.05) were significantly positively correlated with the congruency effect in the triple digit trials. As for the explicit measure of math anxiety, a marginally significant positive correlation was found between maternal help and the sum of children’s responses on the AMAS self-report questionnaire (*r* = 0.28, *p* = 0.07). Mothers’ self-reports of their math skills in high school, however, were significantly negatively associated with children’s math anxiety as measured by the AMAS (*r* = -0.33, *p* < 0.05).

Consistent with the third hypothesis, mothers’ math anxiety was found to have a significant negative correlation with children’s intrinsic math motivation (*r* = -0.32, *p* < 0.05) and a marginally significant negative correlation with intrinsic motivation as a general orientation toward school learning (*r* = -0.30, *p* = 0.06). Mothers’ math course anxiety, however, was found to have significant negative correlations with both intrinsic math motivation (*r* = -0.43, *p* < 0.025) and intrinsic motivation as a general orientation toward learning (*r* = -0.36, *p* < 0.05). For higher levels of mothers’ math anxiety, their children showed not only less intrinsic motivation toward school and math learning, but also poorer arithmetic skills. Mothers’ numerical task anxiety was significantly negatively related to children’s performance in the calculation subtest (*r* = -0.43, *p* < 0.025) and marginally significantly related to their performance in the math fluency subtest (*r* = -0.28, *p* = 0.07). Marginally significant correlations were also found between mothers’ math anxiety as a composite of the three other components (i.e., math test anxiety, numerical task anxiety, and math course anxiety) and children’s scores in the calculation subtest (*r* = -0.30, *p* = 0.06).

As expected, mothers’ math anxiety was found to have a different relationship with explicit and implicit measures of children’s math anxiety. While the correlation between mothers’ math anxiety and children’s math anxiety as measured by the AMAS self-report questionnaire was found to be non-significant (*p* > 0.18), higher levels of numerical task anxiety among mothers were related to a larger congruency effect in the single digit trials in the numerical dot-probe task (*r* = 0.47, *p* < 0.01). This means that children of mothers who report higher levels of math anxiety show more attentional bias toward math-related stimuli compared to children of mothers who report lower levels of math anxiety.

### Predicting Math Anxiety

**FIGURE 6:**
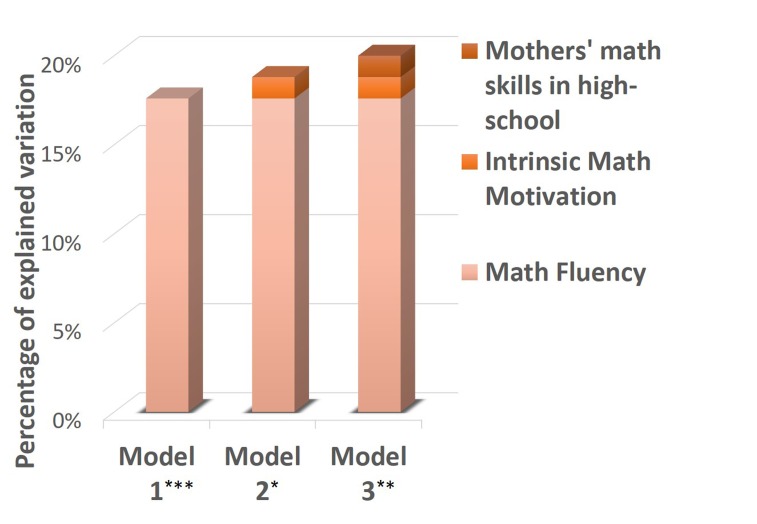
A schematic summary of the hierarchical regression analyses to predict children’s explicitly measured math anxiety (i.e., by the AMAS self-report questionnaire). ^∗^*p* = 0.08, ^∗∗^*p* = 0.05, ^∗∗∗^*p* < 0.05.

**FIGURE 7:**
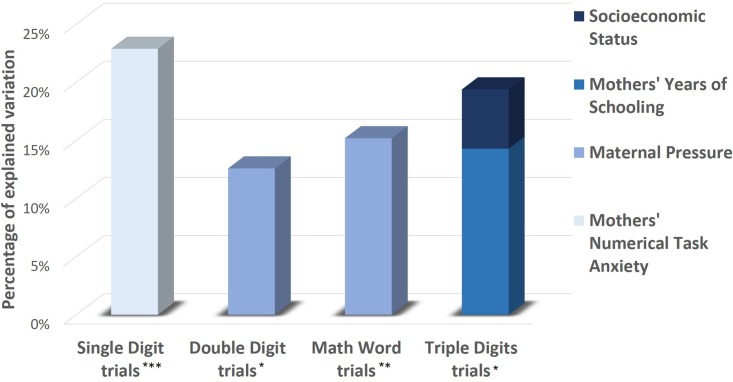
A schematic summary of the hierarchical regression analyses to predict children’s implicitly measured math anxiety (i.e., by the numerical dot-probe task). ^∗^*p* ≤ 0.07, ^∗∗^*p* < 0.05, ^∗∗∗^*p* < 0.025.

For the purpose of identifying the variables with the strongest effect on children’s math anxiety, hierarchical regression analyses were conducted to predict math anxiety as measured explicitly (i.e., by the AMAS self-report questionnaire; **Table 4**) and implicitly (i.e., by the numerical dot-probe task; **Table 5**). **Figures 6** and **7** present a schematic summary of these regression analyses. Each analysis was examined for problems with multicollinearity with the variance inflation factor, but none of the analyses revealed significant problems (largest variance inflation factor = 1.62; Hair et al., 1998; Coakes, 2005). It should be emphasized, however, that caution should be taken when interpreting the regression analyses because of the relatively small sample size.

**Table 4:** Summary of the hierarchical regression analyses to predict children’s explicitly measured math anxiety (i.e., by the AMAS self-report questionnaire).

Predictors	β	*R*^2^	Δ*R*^2^	*F*	*p*
**Step 1**					
Math fluency	-0.42	0.17	0.17	5.34	0.02
**Step 2**					
Math fluency	-0.34	0.18	0.01	2.77	0.08
Intrinsic math motivation	-0.13				
**Step 3**					
Math fluency	-0.38	0.28	0.09	3.01	0.05
Intrinsic math motivation	-0.04				
Mothers’ math skills in high school	-0.31				


**Table 5:** Summary of the hierarchical regression analyses to predict children’s implicitly measured math anxiety (i.e., by the numerical dot-probe task).

Dependent	Predictors	β	*R*^2^	Δ*R*^2^	*F*	*p*
Congruency effect – single digit	Mothers’ Numerical Task Anxiety	0.47	0.22	0.22	7.43	0.01
Congruency effect – double digit	Maternal pressure	0.35	0.12	0.12	3.58	0.07
Congruency effect – triple digit	Step 1					
	Mothers’ Years of Schooling	0.37	0.14	0.14	4.18	0.05
	Step 2					
	Mothers’ Years of Schooling	0.27	0.19	0.05	2.88	0.07
	ISEI	0.24				
Congruency effect – math word	Maternal pressure	-0.38	0.15	0.15	4.46	0.04


*Congruency effect, the difference between reaction time in the incongruent and congruent conditions in the numerical dot-probe task (Rubinsten et al., 2015); ISEI, the International Socioeconomic Index (Ganzeboom and Treiman, 1996).*

First, the explicit measure of math anxiety was used as the dependent variable, entering math fluency scores in the first step, intrinsic math motivation in the second step, and mothers’ self-report of their math skills in high school in the third step. In these analyses, math fluency scores significantly explained 17.6% of the variance [*F*(1,25) = 5.34, *p* < 0.05], such that higher arithmetic skills were associated with lower math anxiety. The contribution of intrinsic math motivation and mothers’ self-report of their math skills in high-school to the regression models was only marginal (for intrinsic math motivation *p* = 0.08; for mothers’ math skills in high-school *p* = 0.05).

Secondly, the implicit measure of math anxiety was used as the dependent variable. Mothers’ numerical task anxiety explained 22.9% of the variance [*F*(1,25) = 7.43, *p* < 0.025] in the congruency effect in the single digit trials. Maternal pressure explained 15.2% of the variance in the congruency effect in the math word trials [*F*(1,25) = 4.46, *p* < 0.05] and marginally significantly explained 12.6% of the variance in the congruency effect in the double-digit trials [*F*(1,25) = 3.58, *p* = 0.07]. To predict the congruency effect in the triple digit trials, mothers’ years of schooling were entered in the first step and socioeconomic status was entered in the following step. In these analyses, mothers’ years of schooling marginally significantly explained 14.3% of the variance [*F*(1,25) = 4.18, *p* = 0.05]. The contribution of socioeconomic status to the regression model was only marginal (*p* = 0.07).

In conclusion, the findings indicated cognitive factors (i.e., arithmetic skills) as better predictors of explicitly measured math anxiety, while environmental factors, and specifically mothers’ numerical task anxiety and maternal pressure, predicted implicitly measured math anxiety.

## Discussion

Math anxiety is a multifaceted phenomenon that arises early in children’s learning (Young et al., 2012; Ramirez et al., 2013; Maloney et al., 2015) due to a combination of environmental (Harper and Daane, 1998), cognitive (Newstead, 1998), and personal factors (Tapia and Marsh, 2004). Yet, the simultaneous relationship between these factors has not previously been examined within an empirical framework. Moreover, the literature on the etiology of math anxiety is mostly based on explicit assessment (Dowker et al., 2016), such as the MARS (Richardson and Suinn, 1972). The present study addressed this knowledge gap by examining how math anxiety is influenced by mothers’ math anxiety and maternal behaviors (environmental factors), children’s arithmetic skills (cognitive factors), and intrinsic math motivation (personal factor). In line with the hypotheses, the results demonstrate that: (a) higher intrinsic math motivation is associated with greater arithmetic skills, and these two constructs have negative correlations with math anxiety; (b) maternal practices with more rigid aspects are related to poorer arithmetic skills, but not to less intrinsic motivation as expected; (c) when the levels of mothers’ math anxiety are higher, their children show less intrinsic motivation, poorer arithmetic skills, and higher levels of implicitly measured math anxiety; and (d) different factors predict accessible self-representations as compared to inaccessible cognitive structures of children’s math anxiety.

In order to rigorously examine children’s math anxiety, both explicit (i.e., the AMAS self-report questionnaire) and implicit tools (i.e., the numerical dot-probe task) were used to assess accessible self-representations of math anxiety as well as inaccessible cognitive structures that are activated automatically. Accordingly, the tendency of the cognitive system of math-anxious individuals to selectively favor the processing of math-related stimuli, which are cognitively interpreted as threatening (Rubinsten and Tannock, 2010), was used as a tool for implicit assessment of math anxiety. This tendency, a phenomenon known as attentional bias (MacLeod et al., 1986; Bar-Haim et al., 2007), was reflected in a higher congruency effect in the numerical dot-probe task (Rubinsten et al., 2015), manifested as faster responses to a probe presented in the same location of a numerical prime as compared to the response time when the probe and numerical prime appeared in different locations. Indeed, one of the key findings highlights the need to evaluate math anxiety by using both explicit and implicit tools. Children’s accessible self-representations of their math anxiety, as reflected by the explicit AMAS self-report questionnaire, were strongly affected by cognitive factors (i.e., arithmetic skills; **Figure 6**). However, children’s implicit perception of their math anxiety, as reflected by an attentional bias in the numerical dot-probe task, was strongly affected by environmental factors (i.e., mothers’ behaviors and attitudes toward math; **Figure 7**).

The need to assess math anxiety through explicit and implicit measures is more pronounced in light of the lack of significant correlations between these tools. Although Rubinsten et al. (2015) found a significant positive correlation between the numerical dot-probe task (i.e., implicit measure) and an explicit tool of math anxiety, they used the Hebrew translated computerized version of the MARS-R (Plake and Parker, 1982) as compared to the AMAS used in the current study for explicit assessment. These contradictory findings may suggest that different self-report questionnaires appraise distinct accessible self-representations of math anxiety. For example, the sMARS (Alexander and Martray, 1989), used in the present study to assess mothers’ math anxiety, focuses on three factors: math test anxiety, numerical task anxiety, and math course anxiety. In contrast, the AMAS (Hopko et al., 2003) consists of nine items representing an index of math anxiety. Indeed, the assessment of each factor of the sMARS separately is of great importance to the present findings. For instance, although mothers’ numerical task anxiety influences children’s implicitly measured math anxiety, mothers’ math anxiety as a composite of the three other components is correlated neither with explicit nor with implicit measures of children’s math anxiety. Thus, various components of children’s explicit perception of their math anxiety may be differentially related to children’s implicit perception of their math anxiety. This issue requires further study. Another explanation may be the relatively small sample in the current study.

### Personal and Cognitive Factors of Math Anxiety

Consistent with the existing literature (Gottfried, 1990; Hembree, 1990; Ma, 1999; Murayama et al., 2013), the results demonstrate that individuals who report high levels of explicitly measured math anxiety (i.e., by the AMAS self-report questionnaire) tend to show low math motivation and poor math performance. Nonetheless, arithmetic skills were the strongest predictor of children’s explicitly measured math anxiety. These findings support previous evidence indicating math anxiety as a result of poor math performance (Ma and Xu, 2004; Maloney et al., 2010; Passolunghi, 2011; Rubinsten et al., 2012; Ramirez et al., 2013; Núñez-Peña and Suárez-Pellicioni, 2014), but they do not rule out the possibility that math anxiety can also be interpreted as a reason for lower arithmetic skills.

Whatever the directionality of the relationship, the links between math anxiety, intrinsic math motivation, and arithmetic skills are supported in the current study. Math anxiety is accompanied by negative emotional, cognitive, and behavioral manifestations, which increase the avoidance of math and math-related situations (Krinzinger et al., 2009). This avoidance behavior creates gaps in math learning that limit future career and earning opportunities (Hembree, 1990; Ashcraft, 2002; Beilock and Maloney, 2015). Furthermore, avoidance of math-related situations may also impair math-anxious individuals’ well-being by arousing symptoms of math anxiety during their engagement in everyday math-related situations, such as calculating a tip at a restaurant or counting change (e.g., Maloney and Beilock, 2012).

Surprisingly, implicitly measured math anxiety (i.e., by the numerical dot-probe task) was not significantly correlated with intrinsic math motivation and arithmetic skills. One can find these results encouraging, because they may indicate the mutability of children’s explicit, conscious, and accessible self-representation of math anxiety. This means that the relationships between cognitive and personal factors and math anxiety were not manifested when evaluating inaccessible cognitive structures that are activated automatically and are thus more stable and robust. Instead, these relationships were reflected only in explicit, conscious, and accessible self-representations of math anxiety. Therefore, explicit, conscious, and accessible self-representations of math anxiety can be moderated through interventions aimed at promoting intrinsic math motivation and achievements, which in turn will change children’s beliefs and attitudes toward math learning.

Another interesting finding suggests the salience of math in the construct of intrinsic motivation toward school learning. According to Gottfried (1990), this salience of math can arise due to the requirement to specialize in a unique symbol system that differs from the verbal processes relied on predominantly in the other subjects. Another explanation may be the tendency to isolate math from other subject areas in elementary school (Stodolsky, 1985). Overall, the findings indicate the importance of intrinsic math motivation in the development of math anxiety and academic success. The critical role of intrinsic math motivation was emphasized in previous studies (Lyons and Beilock, 2011; Wang et al., 2015), indicating the importance of motivating behaviors in mitigating the effects of math anxiety on math performance. This evidence may be perturbing in view of the documented decline in intrinsic math motivation from elementary to high school (e.g., Corpus et al., 2009; Garon-Carrier et al., 2016).

### Environmental Causes of Math Anxiety

Another aim of the current study was to investigate the role of mothers’ behaviors and attitudes toward math in the development of children’s math anxiety. First, environmental factors were found to be the strongest predictors of children’s implicitly measured math anxiety (i.e., by the numerical dot-probe task). Generally, these findings support the common assumption about the major contribution of parents to their children’s attitudes, interests, and education (Fan and Chen, 2001; Koutsoulis and Campbell, 2001; Bong, 2008; Prins and Toso, 2008). The effects of mothers’ behaviors and attitudes toward math on children’s math anxiety appeared to be unconscious, as they were reflected in cognitive structures of math anxiety that are inaccessible to the individual and activated automatically. This affective automatic processing represented the tendency of anxious individuals to display an attentional bias toward threat-related information relative to neutral stimuli (MacLeod et al., 1986; Bar-Haim et al., 2007). A differential attentional allocation toward threat-related information has been found to have a prominent role in the development and perseverance of anxiety disorders (Mathews and MacLeod, 2002).

Evidence of the existence of various mother-related predictors of implicitly measured math anxiety may imply the benefit of using the numerical dot-probe task. Different environmental factors influenced children’s attentional bias depending on the difficulty levels of the math-related stimuli displayed. Whereas maternal pressure predicted attentional bias in the math word trials, mothers’ numerical task anxiety predicted attentional bias in the single digit trials. This can be explained by previous findings indicating smaller working memory spans among math-anxious individuals than among people with less math anxiety, particularly in complex tasks that require mental calculation and keeping numbers in memory simultaneously, such as the equation trials (Ashcraft and Kirk, 2001; Young et al., 2012). Therefore, it seems that various environmental factors affect different levels of anxiety, which are reflected in the difficulty levels of the math task.

The negative contribution of maternal pressure to children’s math anxiety is in line with the well-documented relationship between parental behaviors and children’s anxiety-related behaviors in general (e.g., Wood et al., 2003; McLeod et al., 2007; Quach et al., 2015). In the field of math anxiety, it has been shown that with higher parental involvement in children’s math-learning, children were less math anxious (Roberts and Vukovic, 2011) and performed better on higher levels of math (Vukovic et al., 2013). Conversely, the present study indicated poorer arithmetic skills among children of mothers who are more involved in their children’s learning by exerting pressure to retain high levels of performance, helping with schoolwork, stress for intellectual development, and monitoring.

One possible explanation of these contradictory findings may be the cultural differences between the populations studied. For example, the definition of parental practices, such as the characterization of the amount of help given to a child with homework as high or low, may vary in different cultures. Likewise, no significant correlations were found between parenting practices and intrinsic math motivation, although the literature established a positive contribution of psychologically supportive atmosphere at home (Koutsoulis and Campbell, 2001; Campbell and Verna, 2007; Bong, 2008; Fan and Williams, 2010) and a negative contribution of parental pressure (Ginsburg and Bronstein, 1993; Bong, 2008), help and monitoring (Campbell and Verna, 2007) to children’s intrinsic math motivation. These results highlight the need to define and specify in future studies the precise manner in which parents are involved in children’s learning.

As for the effect of mothers’ math anxiety on their children’s beliefs and attitudes toward math, the findings suggest intergenerational transmission of math anxiety. Similarly, Maloney et al. (2015) indicated that children of math-anxious parents acquired significantly less math knowledge during the school year and demonstrated higher levels of math anxiety at the end of the school year, but only if math-anxious parents frequently help their children with math homework. However, there is a fundamental difference between this study and the current research. While the first assessed children’s math anxiety explicitly (i.e., by a self-report questionnaire), the effect of mothers’ math anxiety in general, and numerical task anxiety in particular, on the development of children’s math anxiety in the current study was demonstrated only in the implicit assessment. Thus, the direct transmission of math anxiety may be manifested in children’s inaccessible cognitive structures, whereas the influence of parents’ math anxiety on children’s self-representations of math anxiety depends on the extent to which parents transfer their negative conception of math to their children.

Further, the results emphasize the crucial role of mothers’ attitudes toward math in the development of their children’s attitudes toward learning, math-learning, and even achievements. Moreover, maternal socio-demographic characteristics correlated with children’s implicit perception of their math anxiety, as reflected by an attentional bias in the numerical dot-probe task, especially at higher levels of arithmetic, such as the triple digit trials. These findings are in line with previous studies (Turner et al., 2002; Scarpello, 2007) and may imply that children of parents with reduced educational experiences and limited economic resources tend to have negative attitudes toward learning in general and math-learning in particular. Therefore, more attention should be devoted to these children who may be at risk for developing math anxiety.

In sum, the contribution of the current research to the existing literature lies in addressing several knowledge gaps. First, the simultaneous relationship between environmental, cognitive, and personal factors of children’s math anxiety has not yet been examined. The findings demonstrate that in order to better understand parents’ role in their children’s math anxiety, multiple facets of parenting and of the children’s math skills should be taken into consideration. Secondly, this study explored the etiology of math anxiety by using both explicit and implicit measures of math anxiety. On the one hand, children’s conscious and accessible self-representations of math anxiety, as reflected by the explicit self-report questionnaire, are strongly affected by cognitive factors. On the other, children’s implicit and unconscious cognitive constructs of math anxiety, as reflected by an attentional bias in the numerical dot-probe task, are strongly affected by environmental factors. Furthermore, the present study provides preliminary evidence of an intergenerational transmission of math anxiety.

### Limitations

Although the current study makes novel contributions to the existing literature, there are several limitations. First, norms regarding parenting practices may vary between cultures. These potential cultural differences are not captured in this study, as mentioned above. Second, the sample size and the correlational and cross-sectional data limit the generalizability of the findings. The relatively small sample size also limits interpretations regarding causality, and caution is required. Therefore, longitudinal data are needed to identify possible causal relationships. The present study can serve as a pilot study and help future studies by implying a trend of causality, which should be interpreted, as noted, with caution. Third, only mothers participated in the study, limiting information about the gender of the parent, which might be influential. It may be argued that the parent participating may be the one most involved in children’s education, but this assumption is tenuous. Additionally, socioeconomic status will be more reliable when measured by the occupation of both parents rather than only by the mother’s occupation, as in the present study. Future research should consider gathering information from both parents. Moreover, future research could receive more comprehensive information about parents’ influences on children’s math anxiety through observation of parental behaviors in a natural environment. In addition, it would be interesting to examine parents’ math anxiety by using both explicit and implicit measures, especially given the contribution of this rigorous and comprehensive assessment among children in the current study.

### Implications

Despite the reviewed limitations, the present findings contribute to the literature and hold practical implications for parents, educators, psychologists, and policy makers seeking to reduce the prevalence of math anxiety among students. Different factors predicted children’s math anxiety based on how anxiety was assessed (i.e., explicit vs. implicit measures of math anxiety). Thus, it might be necessary for future research to examine math anxiety through both explicit measures, such as the AMAS self-report questionnaire, and implicit tools, such as the numerical dot-probe task. Notably, use of the numerical dot-probe task enables exploration of different levels of math anxiety by manipulating the complexity of the task.

For educators, the results highlight the value of fostering intrinsic math motivation among children. School-based interventions designed to promote this construct appear to be particularly important, not only for improving math achievements, but also for reducing math anxiety. Innovative programs and curricula aimed at reinforcing children’s intrinsic math motivation should target early childhood education because of the documented decline in this construct from elementary to high school (e.g., Corpus et al., 2009; Garon-Carrier et al., 2016). Furthermore, from the age of 9 years, the levels of intrinsic math motivation in each school year have been found to predict the levels in the following school year (Gottfried et al., 2001).

The valuable findings in the current study that require special attention by parents and school psychologists refer to the major role of parents in the development of children’s beliefs and attitudes toward learning in general and math in particular. Parents’ math anxiety, parenting with more controlling aspects, and parental involvement in children’s learning were related to higher levels of math anxiety, poorer arithmetic skills, less intrinsic math motivation, and lower levels of intrinsic motivation toward school learning. To this end, school psychologists could conduct informative sessions for parents to praise the benefits of becoming more empathetic, autonomous, and psychologically supportive in their parenting practices.

Educators and policy makers should also take into account the relationship between socio-demographic characteristics, such as parents’ educational level and socioeconomic status, and math anxiety. In order to address this issue, there is a need to invest more resources in schools in low socioeconomic communities. These resources should be used as efficiently as possible to promote interventions aimed at reducing math anxiety and its effects among students.

## Conclusion

The current study emphasizes the importance of assessing math anxiety by using both explicit and implicit tools. Through this rigorous and comprehensive assessment, the results demonstrate the influence of arithmetic skills on sixth-grade children’s explicit, conscious, and accessible self-representations of math anxiety, as manifested by the AMAS self-report questionnaire. However, the crucial role of mothers’ behaviors and attitudes toward math is more substantial in the prediction of children’s implicit and unconscious cognitive structures of math anxiety, as manifested by an attentional bias in the numerical dot-probe task. Furthermore, the present study provides preliminary evidence of intergenerational transmission of math anxiety. Overall, the findings indicate that parents, educators, school psychologists, and policy makers should work together in order to reduce the prevalence of math anxiety and its effects in elementary school.

## Author Contributions

LDC was the lead author in conceptualizing the research and writing the manuscript. OR contributed to all steps of the research and then critically reviewed and revised the manuscript. All authors accepted accountability for the final version of the manuscript.

## Conflict of Interest Statement

The authors declare that the research was conducted in the absence of any commercial or financial relationships that could be construed as a potential conflict of interest.
